# VISPA: a computational pipeline for the identification and analysis of genomic vector integration sites

**DOI:** 10.1186/s13073-014-0067-5

**Published:** 2014-09-03

**Authors:** Andrea Calabria, Simone Leo, Fabrizio Benedicenti, Daniela Cesana, Giulio Spinozzi, Massimilano Orsini, Stefania Merella, Elia Stupka, Gianluigi Zanetti, Eugenio Montini

**Affiliations:** San Raffaele Telethon Institute for Gene Therapy (TIGET), San Raffaele Scientific Institute, 20132 Milano, Italy; Center for Advanced Studies, Research and Development in Sardinia (CRS4), 09010 Pula, CA Italy; Università degli Studi di Cagliari, 09124 Cagliari, Italy; Department of Informatics, Systems and Communication (DISCo) - University of Milano-Bicocca, Milano, Italy; Center for Translational Genomics and Bioinformatics, San Raffaele Scientific Institute, Via Olgettina 58, 20132 Milano, Italy

## Abstract

The analysis of the genomic distribution of viral vector genomic integration sites is a key step in hematopoietic stem cell-based gene therapy applications, allowing to assess both the safety and the efficacy of the treatment and to study the basic aspects of hematopoiesis and stem cell biology. Identifying vector integration sites requires ad-hoc bioinformatics tools with stringent requirements in terms of computational efficiency, flexibility, and usability. We developed VISPA (Vector Integration Site Parallel Analysis), a pipeline for automated integration site identification and annotation based on a distributed environment with a simple Galaxy web interface. VISPA was successfully used for the bioinformatics analysis of the follow-up of two lentiviral vector-based hematopoietic stem-cell gene therapy clinical trials. Our pipeline provides a reliable and efficient tool to assess the safety and efficacy of integrating vectors in clinical settings.

**Electronic supplementary material:**

The online version of this article (doi:10.1186/s13073-014-0067-5) contains supplementary material, which is available to authorized users.

## Background

Viral vectors, due to their ability to permanently integrate in a target genome, are used to achieve the stable genetic modification of therapeutically relevant cells and their progeny. In particular, γ-retroviral (γ-RVs) and lentiviral (LVs) vectors are the preferred choice for hematopoietic stem/progenitor cell (HSPC) gene therapy (GT) applications, having proved their efficacy in several preclinical assays and clinical trials for inherited monogenic disorders [1-4]. Since γ-RVs and LVs integrate in the cellular genome in a semi-random fashion [5-8], in a population of vector marked cells each clone and its progeny harbor an integrated vector in a unique genomic position that can be used as a distinctive genetic identifier.

Studies aimed at investigating the genomic distribution of integrating vectors in blood cells of GT patients are fundamental to assess the safety and efficacy of the therapy. Indeed, in some cases HSPC-GT was associated to the potential emergence of severe adverse effects that involve the perturbation of the expression of genes in the proximity of the vector’s integration site (IS), a phenomenon known as insertional mutagenesis (IM) [1,9-12]. The identification of ISs on leukemic cells from GT patients and preclinical models allowed identifying the causes of IM and tracking the evolution of the malignant clone over time [11-16]. State-of-the-art strategies for the identification of ISs start with the amplification, via polymerase chain reaction (PCR), of the DNA portion that contains part of the proviral genome and of the flanking cellular genome. PCR products are then sequenced and mapped to the reference host genome to determine the genomic coordinates of the ISs. More recently, next generation sequencing (NGS) approaches have greatly enhanced the power of IS analysis, allowing to recognize clonal expansions caused by *in vivo* selection of gain-of-function insertional mutants even before they progress to overt malignancy. Moreover, IS analysis is useful to address clonal diversity during hematopoietic reconstitution and the levels of HSPC marking and activity after transplant, thus providing readouts for efficacy. For these reasons, over the past years there has been a constant increase in the amount of sequencing and mapping of vector/genomic DNA junctions, as well as an increasing diversification of tissue sources, cell types and time points during IS monitoring. However, despite the advances brought forth by NGS approaches and the higher level of detail provided by the additional cell types and time points, there is still a clear lack of computational tools that offer both a level of performance capable of dealing with the huge amount of data generated by sequencing platforms and sufficient usability to make them accessible to investigators with varying degrees of technological expertise.

Here we describe the design and implementation of VISPA (Vector Integration Sites Parallel Analysis), a bioinformatics pipeline for the identification of ISs built on a scalable infrastructure with a simple graphical user interface (GUI) based on the popular Galaxy framework [17]. VISPA has been successfully applied in several studies on mouse and human genome [3,4,18,19]. In this work we describe its performance on human IS datasets. This analysis allowed us to highlight critical points negatively impacting the efficiency of IS retrieval and mapping and provided hints to improve the whole process. VISPA is available at [20].

## Implementation

**Figure 1:**
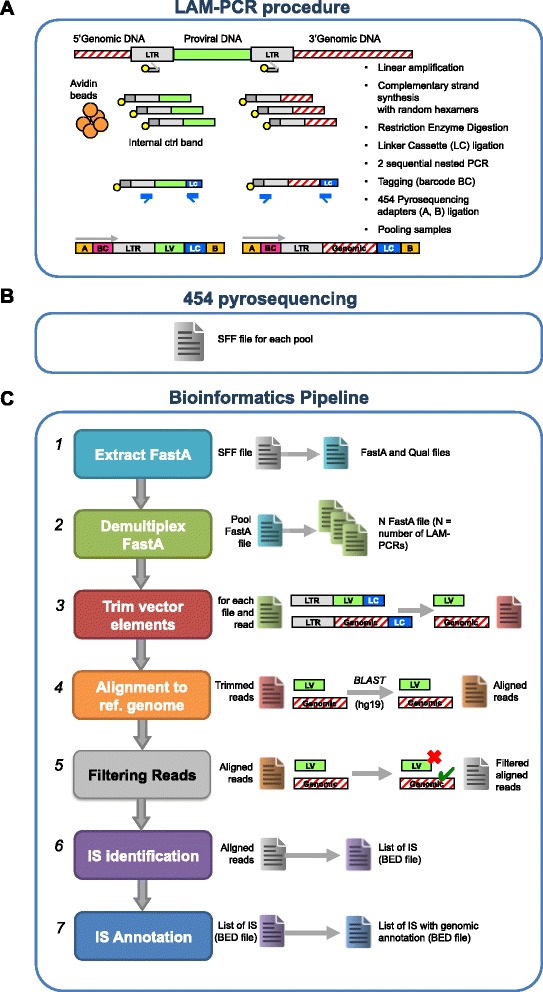
**IS analysis procedure for the Roche 454 sequencing platform. (A)** DNA fragments containing the vector-cellular genome junctions are retrieved and amplified from vector marked genomic DNA by LAM-PCR. **(B)** LAM-PCR products are processed by the NGS platform, yielding sequencing reads that have to be processed *in silico*. **(C)** Bioinformatics pipeline, from FASTA extraction to IS identification and annotation.

VISPA has been specifically designed to analyze DNA fragments generated by linear-amplification (LAM) mediated PCR [21], a technique used to retrieve and amplify DNA fragments containing the junctions between the integrated proviral and the cellular genome. Due to its high sensitivity and accuracy, LAM-PCR is the current standard for preclinical and clinical GT studies. The DNA fragments generated with this method range from 100 to 1,000 bp in length, and contain the proviral long terminal repeat (LTR), the flanking genomic DNA and a linker cassette (LC). LAM-PCR products are then reamplified by PCR with fusion primers containing a specific 6-nucleotide sequence (barcode) that acts as a tag to allow sample recognition after multiplexing. Barcoded fragments are then purified, quantified, grouped into pools and sequenced with either Roche 454 or Illumina MiSeq platforms. As a result of this procedure, the sequencing reads contain not only the genomic fragment needed for IS identification, but also viral and artificial sequences that must be trimmed out before alignment to the reference genome. Finally, sequencing reads must be processed by a bioinformatics pipeline that yields the final list of annotated ISs (Figure 1).

### Bioinformatics pipeline

The bioinformatics pipeline (Figure 1C) consists of several sequential steps that lead from raw sequencing reads to the annotated ISs. The first step converts reads from the output format of the sequencer to the FASTA format; sequencing data are then parsed to identify barcodes and perform demultiplexing (that is, write a separate FASTA file for each barcode); the LTR and LC sequences are subsequently removed from each read to isolate genomic fragments; in the next step, reads are mapped to the reference genome and several filters are applied to ensure unambiguous alignment; after that, ISs that fall in the same 3 bp window are merged together; finally, all ISs are annotated by listing nearby genomic features (for example, genes). In a subsequent postprocessing step, each IS is associated to the LAM-PCR sample from which it was originally derived, allowing its assignment to a source (for example, peripheral blood, bone marrow and so on), cell type (for example, CD4+ T cell, CD19+ B cell, and so on), and time point after treatment.

### Format conversion

In the data extraction step, standard flowgram format (SFF, for the Roche 454) or FASTQ (for the Illumina MiSeq) files are converted to the FASTA format with a wrapper for the sff_extract program [22]. In the course of our experiments, before running the pipeline, we also had to convert paired-end FASTQ files from the Illumina MiSeq to single-strand. In the case of Illumina paired-end data, before running the pipeline, we converted the reads to single-strand as follows: for each read, we determined the LTR’s orientation to identify the starting nucleotide; then, for overlapping reads, we merged the pair, while for non-overlapping reads we only kept the one that contained the LTR, which allows identifying the IS.

### Demultiplexing

To avoid NGS capacity underutilization, several samples are often sequenced at the same time, a technique called multiplexing. To enable the redistribution of output reads into separate groups (demultiplexing), samples are tagged with individual ‘barcode’ sequences. Our demultiplexing tool, implemented in Python, identifies barcodes and uses them to demultiplex sequence data, producing a separate FASTA file for each barcode. To demultiplex sequencing data, we developed a simple exact string pattern matching: the input is a list of barcode sequences that will be searched for at the beginning of each read, while the output consists of a separate FASTA file for each barcode (reads that do not contain any known tag are discarded). To avoid biases due to the possible misclassification of similar sequences, no mismatches are tolerated in this phase.

### Trimming

Sequencing reads produced in the context of GT contain both viral fragments and artificial sequences introduced as a side effect of the procedure. In the trimming step, these sequences (the LTR and LC) are identified and removed to isolate the genomic fragments. Our implementation consists of a Python program that integrates the BLAST [23] alignment engine via a Boost.Python [24] wrapper around the NCBI C++ Toolkit [25]. The program searches for a subsequence consisting of the last 63 nucleotides of the LTR, imposing an alignment homology of at least 89% and a perfect match on the last 3 bases. If the LTR is found, it is removed from the read and the resulting sequence is kept for further analysis to avoid mapping aspecific amplification products. All reads that do not contain the LTR are instead discarded. Reads that pass the above filter are searched for the presence of the LC, which is also trimmed out from the read. Note that the absence of the LC does not imply that the read is the product of an aspecific amplification reaction: thus, reads that do not contain the LC are not eliminated from subsequent analysis, in contrast with what happens to the LTR. Finally, all trimmed sequences less than 20 bp long are discarded. As shown in the Results section, this setup results in a highly accurate identification of the junction point between the LTR and the genomic sequence, which represents the IS itself. Hereinafter, we will refer to the set of trimmed reads as ***T***.

### Alignment and filtering

To find out where in the host DNA the viral vector has integrated itself, sequencing reads must be mapped to a reference genome. Our implementation consists of a distributed version of BLAST with custom filters: for each read in ***T***, BLAST outputs a series of hits corresponding to matching genomic regions, supported by statistics such as alignment score, starting position, and so on. Since an IS is defined as the junction between the vector and the host genome, the part of the sequence flanking the viral LTR must be identified as accurately as possible, and reads must be univocally mapped to the reference genome: these requirements are addressed by a series of filtering procedures described in the rest of this section.

In our experiments, to avoid mapping errors, we discarded all hits with an identity score lower than 95% as well as those with a starting alignment position beyond the third base. The rationale behind the latter filter is justified by PCR biases and biological changes for which the position of a given IS can oscillate in a range of +/- 3 bases with respect to aligned reads [26,27]. Although these are the recommended choices, both the minimum identity score and the maximum starting position are exposed as configurable parameters to the user. In the following text, reads successfully mapped to the reference genome according to the above rules will be denoted as ***M***, while discarded reads will be referred to as ***N***, so that |***T***| *=* |***M***| + |***N***|.

In order to univocally associate a genomic region to each IS, sequencing reads that map equally well to multiple regions of the genome must be discarded: thus, reads from ***M*** are subject to further filtering in order to isolate unambiguous alignments. We here introduce the homology score ***hs*** that, for every mapping ***m*** of a given read ***i***, represents the percentage of aligned bases with respect to the length of the read:

$$ \mathrm{h}{s}_{\mathrm{im}} = 100 \times {\left|{q}_s-{q}_e\right|}_{im}/{l}_i $$

where *q*_*s*_ and *q*_*e*_ are, respectively, the starting and ending positions of the query (input read) in the alignment as reported by BLAST, and *l* is the length of the read. A read is classified as unambiguously aligned (***U*** set) if its best hit in terms of alignment score ***as*** has significantly better values of both ***as*** and ***hs*** than the second best hit; otherwise, it is discarded as ambiguously aligned (***A*** set, so that |***T***| = |***U***| + |***A***| + |***N***|). More specifically:

1. All hits for a given read are sorted in decreasing ***as*** order ***as***_***(1)***_, ***as***_***(2)***_, … ***as***_***(|M|)***_;
2. If both |***as***_***(2)***_ - ***as***_***(1)***_| > ***as***_***t***_ and |***hs***_***(2)***_ - ***hs***_***(1)***_| > ***hs***_***t***_, where ***as***_***t***_ and ***hs***_***t***_ are predefined thresholds, the alignment is classified as unambiguous. In our experiments, we set ***as***_***t***_ and ***hs***_***t***_, respectively, to 15 and 20, after a parameter tuning phase performed on a controlled murine dataset.

Since the two LTRs present in the integrated proviral form of LV are direct repeats, LAM PCR amplification also generates a product containing part of the lentiviral genome downstream the 5’ LTR. To detect viral sequences, we added a ‘dummy chromosome’ to the reference genome, corresponding to the vector genome: in this way, NGS reads are also aligned to the vector genome, and reads that map to both genomes are shown in the BLAST output, enabling their removal from subsequent steps. Finally, we applied an alignment quality filter to the reads in ***U***, discarding all alignments with a BLAST identity score lower than 95% and ***hs*** less than 80%. The set of reads that pass this filter will be subsequently denoted as ***R***.

### Integration site merging

Due to the possible presence of technical biases, we applied a previously validated [6,26,27] 3 bp tolerance window on the genomic position of the IS (that is, the starting point of the alignment): all reads in ***R*** that lie in the same window are merged into a single locus, represented by the first position in the window itself. This is achieved by simply sorting reads by their starting position on each reference chromosome and running a sliding window [28] on the sorted list. We will refer to the resulting set of distinct IS as ***L***.

### Integration site annotation

The final step is the *annotation* of ISs, where each site is associated to nearby genomic features such as genes, miRNAs, and so on. We developed our own annotation tool that takes as input two main parameters:

1. The ***L*** set, with each IS characterized by (at least) its genomic location, that is, chromosome name and position within the chromosome;
2. A browser extensible data (BED) file [29] containing a list of genomic features, each characterized by (at least) its name, the name of the chromosome on which it is found, its starting and ending position on the chromosome itself, and its orientation (plus or minus strand). Examples of gene annotation BED files are available in our Galaxy front-end in the shared library area.

For each IS, the program finds the closest feature(s) among those listed in the annotation file and, for each feature, outputs the following information: the (chromosome, position) tuple that identifies the IS; the name and strand of the feature as they appear in the BED file; the feature’s starting and ending position; the distance of the IS from the feature’s transcription start site (TSS); the relative position of the IS with respect to the feature (upstream, downstream or in-gene); the integration percentage for in-gene integrations (from 0% when the IS coincides with the TSS to 100% when the IS lies at the opposite end of the feature).

### Development

All tools in the pipeline were developed in Python, used either exclusively or as a wrapper around foreign libraries and external executable. For each tool, we built a Galaxy [17] front-end that allows interacting with it through an intuitive interface based on text boxes, drop-down menus, and so on.

**Figure 2:**
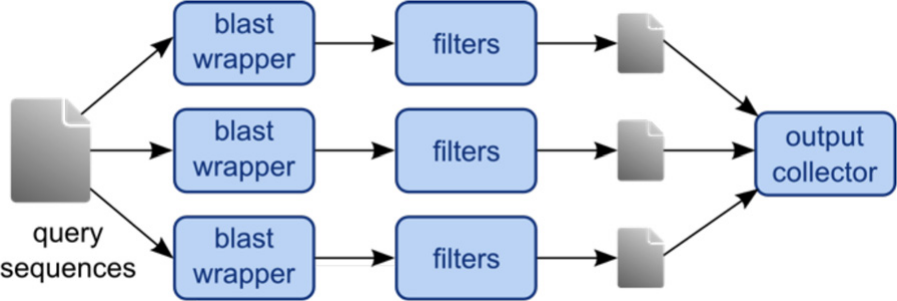
**Simplified architecture of the distributed alignment, filtering tool, control, and flow in the distributed implementation.** The MapReduce workers repeatedly call BLAST on each query sequence in the input subset assigned to it by the Hadoop framework. Each stream of BLAST results is then filtered according to the specified rules: if there are no results left at this point, the read is discarded (*N* set, no-hit); otherwise, remaining hits are classified as either ambiguous (*A* set, repeats) or unambiguous (*U* set). Finally, a local output collector opens all MapReduce output files (one per worker) and merges them into three new files, one for each category. In the course of the data analysis performed for our clinical trials, the alignment and filtering step has been run on up to 240 CPU cores simultaneously.

With the exception of the alignment and filtering step, all programs have been implemented as ordinary executable scripts depending on a common software library. The alignment and filtering step, on the other hand, posed a significantly greater challenge in terms of running time and scalability. In our experiments, nearly 14 million input reads had to be mapped to the whole human reference genome: a task that, on a single processor, would have taken an amount of time incompatible with the turnaround requirements of the clinical trials. Since the mapping job is easily parallelizable on the set of input sequences and a near-linear speedup can be achieved by partitioning the input dataset set into a number of subsets equal to that of available CPU cores (in the ideal case of perfect load balancing), we implemented the tool as an application for Apache Hadoop [30], a distributed computing framework that handles dataset partitioning, load balancing, and re-execution of failed task transparently according to the MapReduce paradigm [31]. While Hadoop’s native API is in Java, to keep the code base consistent with the rest of the pipeline we developed the application with Pydoop [32], a Python API for Hadoop developed at CRS4 (Figure 2).

From an architectural standpoint, the pipeline is structured as follows:

- A graphical web-based user interface built with Galaxy;
- A job dispatcher and workflow manager, also based upon Galaxy;
- A high-performance computer cluster where applications are actually run.

**Figure 3:**
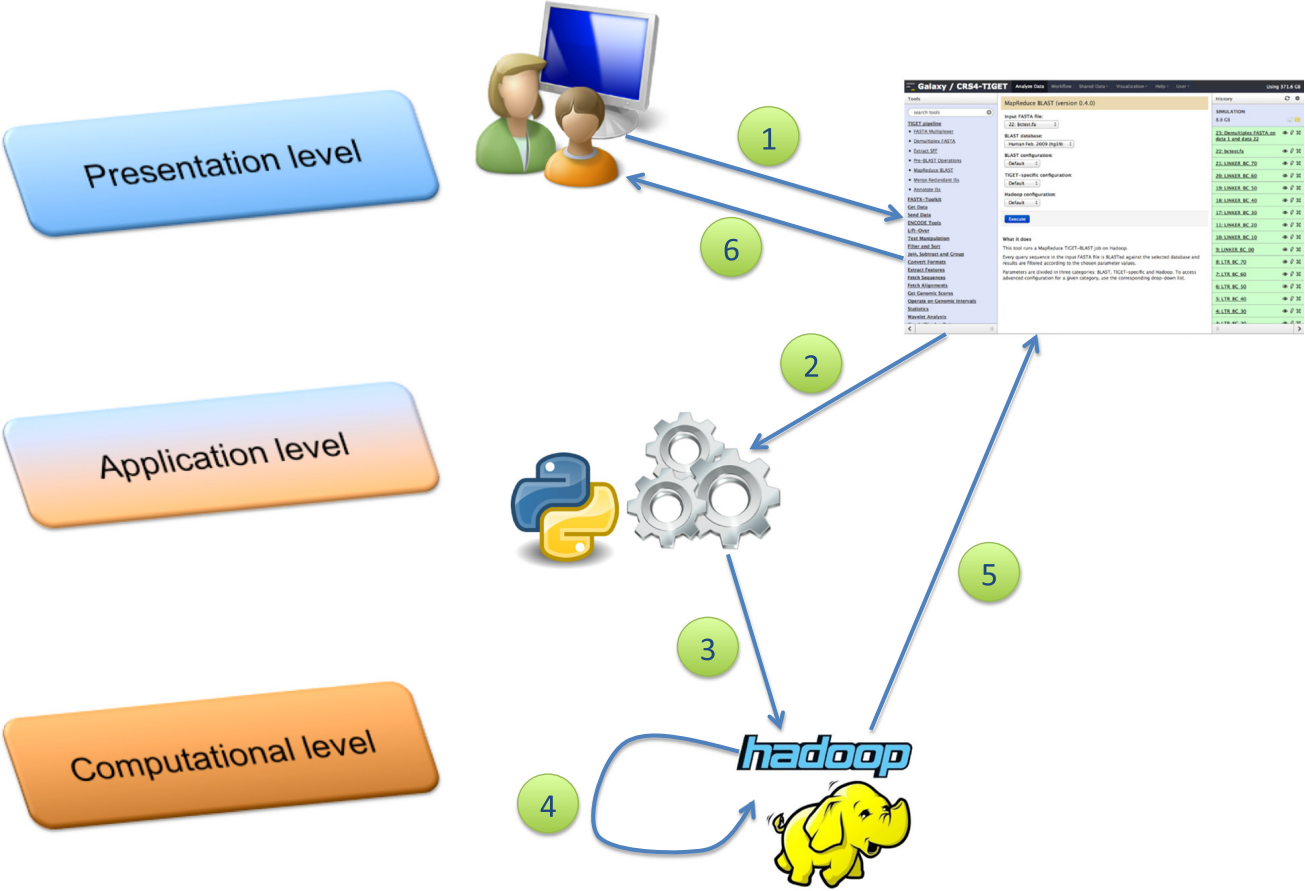
**Schematic representation of the pipeline’s architecture.** Interaction with the system happens through a Galaxy-based GUI: after logging in to the web site, the user is able to upload data and customize the various parameters using a dedicated subsection of the Galaxy tool panel **(1)**. Although each pipeline step can be run as a stand-alone tool, Galaxy also allows to combine them in a single workflow, thus enabling the automation of the entire process. When the user submits a job, Galaxy transfers all parameters chosen via the GUI to a driver script **(2)**, which schedules the actual computation on the computing cluster **(3)**. In the case of the alignment and filtering step, actual processing is further delegated to Hadoop **(4)**, while the driver script acts as a final output collector. Finally, job output is returned to the graphical front end **(5)** that presents final results to the user **(6)**.

The Galaxy server runs on a node enabled for job submission on the cluster’s resource manager (RM). While most pipeline tools are executed on single CPU cores assigned to them by the RM, the distributed alignment and filtering step runs concurrently on cluster subsections managed by Hadoop (Figure 3).

## Results and discussion

We performed a series of tests to evaluate VISPA’s reliability in IS identification as well as its performance in the analysis of large datasets. We generated an *in silico* dataset of IS and used it to test our tool and other publicly available software for IS analysis, as described in the next subsection. In the following subsection, we characterized the computational performances of our tool by analyzing large datasets of real IS previously obtained from two GT studies [3,4].

### Reliability of VISPA and other tools for IS analysis

**Figure 4:**
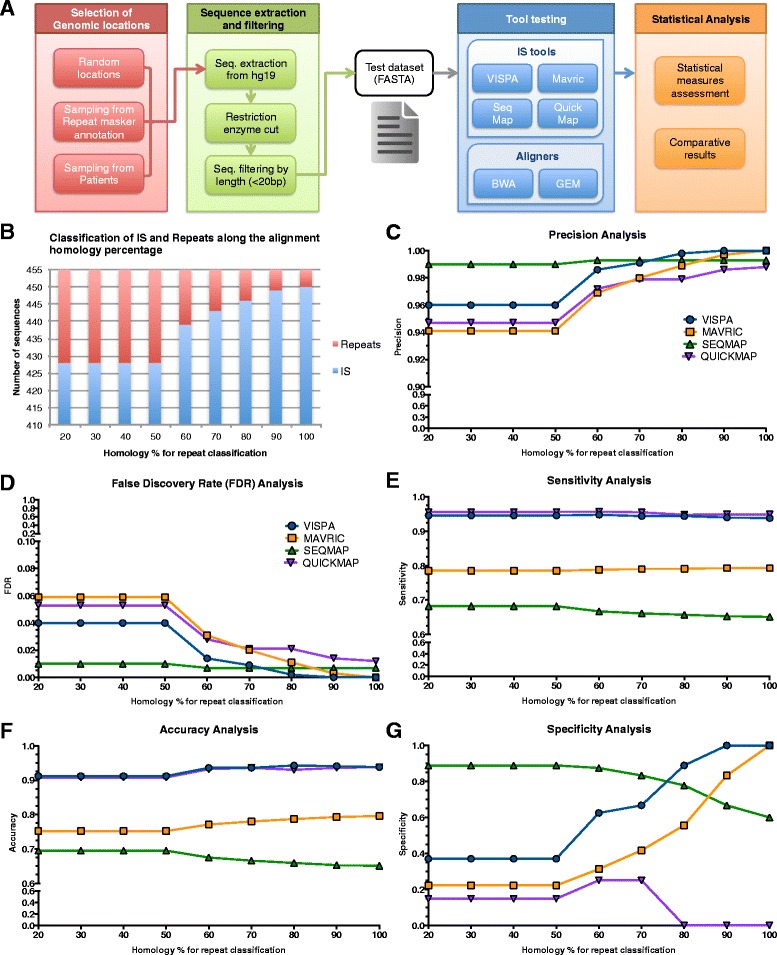
**Reliability evaluation of VISPA and other IS analysis tools. (A)** Overall strategy for reliability assessment, from the generation of the synthetic dataset to the final results. **(B)** Number of sequences classified as ISs or repeats for increasing homology percentage thresholds. **(C-G)** Precision, FDR, sensitivity, accuracy, and specificity of all tested tools for increasing homology percentage thresholds.

We assessed the reliability of our tool and other available software (Mavric [33], SeqMap [34] and QuickMap [35]), on an *in silico* dataset of 455 human sequences that simulate ISs with pre-determined genomic coordinates, characterized by different length, sequence complexity and mappability (see Additional files 1 and 2). We analyzed this dataset with VISPA and the other selected tools comparing results with the expected outcome (see strategy in Figure 4A). We exploited two of the most recent next generation sequencing (NGS) aligners, BWA [36] and GEM [37], as reference to verify the mappability of the test sequences on the target genome, thus allowing the classification of each sequence as a repeat or not. The classification of each sequence as repeat or unique position in the genome can be computed using a ratio between the alternative/suboptimal alignment score and the optimal one (for simplicity here called homology percentage or ratio). Once an appropriate threshold has been set for the homology ratio, input ISs are either accepted as unambiguously mapped or rejected as repeats. Figure 4B shows how classification results change as the homology percentage threshold varies between 20% and 100%, thus simulating a varying degree of stringency. For instance, in our synthetic dataset, a 90% threshold (that means that two alignments are considered repeats if and only if the ratio the alternative alignment and the optimal one is ≥ 0.9) leads to 449 accepted and six rejected ISs. For all four tools, sequences passing homology filtering were subsequently labeled as discarded (not identified as a mapped IS), correctly matched (if the chromosome and genomic position are correct within +/- 2 bp), or mismatched (wrong chromosome and/or genomic position). In this framework we are able to use standard statistical measures to evaluate precision, sensitivity, specificity, accuracy, and false discovery rate (FDR) by accounting for what we expect to observe in our data (given an homology percentage ratio, or testing for an increasing value of homology ratio) *versus* what we obtained from the tools. True positives (TP) are actual ISs that are identified as such by a tool; false positives (FP) are actual repeats that are identified as ISs and mismatched ISs; false negatives (FN) are actual ISs identified as repeats; true negatives (TN) are actual repeats (discarded sequences) that are identified as such.

Since the number of classified IS and repeats did not changed up to the homology percentage value of 50 (Figure 4B), we compared statistical results of the IS analysis tools in the neighborhood interval of 70%. In terms of precision (Figure 4C) and FDR (Figure 4D), VISPA and SeqMap resulted the best tools. In terms of sensitivity (Figure 4E), VISPA and QuickMap performed similarly (0.94 and 0.95, respectively) in top ranking positions, whereas MAVRIC and SeqMap achieved lower values (less than 0.8); similar results were obtained for the accuracy (Figure 4F). For the specificity (Figure 4G) we observed for VISPA an increasing trend as the homology percentage increased, reaching 1 at 90% homology; only MAVRIC showed a similar trend, while SeqMap and QuickMap presented an opposite behavior, with the latter reaching 0 at 80% homology. For what concerns the analysis of mismatched IS, MAVRIC yielded an amount of 50 mismatched IS clustered in a distance between 100 and 500 bp from the reference IS position (Additional file 3A; in contrast, SeqMap presented only 1 mismatched IS, located in a different chromosome; finally, QuickMap yielded 18 mismatched ISs, the majority of them (12) within 10 bp from the reference position (Additional file 3B).

### Computational performance of VISPA on IS datasets from GT studies

We analyzed the performance of VISPA in the context of the analysis of 19,306,267 raw sequence reads obtained in two GT previous studies [3,4]. After quality control and barcode filtering, 18,874,038 total input reads were selected, 13,786,956 of which contained a valid LTR sequence (the previously introduced ***T*** set); these reads were subsequently aligned to the human reference genome (build hg19/GRCh37, February 2009) yielding 12,717,773 mappings (corresponding to the ***M*** set) and 1,069,183 unmatched reads (the ***N*** set); after the filtering step, the ***M*** set was further split into the ***A*** and ***U*** subsets, with a total amount of, respectively, 2,572,931 and 6,035,527 reads. LV detected reads were 4,109,315. The alignment quality filter discarded 541,122 reads, leaving a total amount of 5,494,405 redundant ISs (the ***R*** set). After merging ISs according to the sliding window method described above, the resulting 71,359 distinct ISs (the ***L*** set) were finally annotated with nearby genomic features.

From this analysis we found that for all patients, despite the large amount of sequences generated by the 454-Roche or MiSeq Illumina platforms, after the sequential filtering steps applied by our pipeline, the number of sequencing reads univocally mapped on the genome was progressively reduced to 30% of the initial number of sequencing reads. Several reasons, although to a different extent, concurred to this strong reduction.

**Figure 5:**
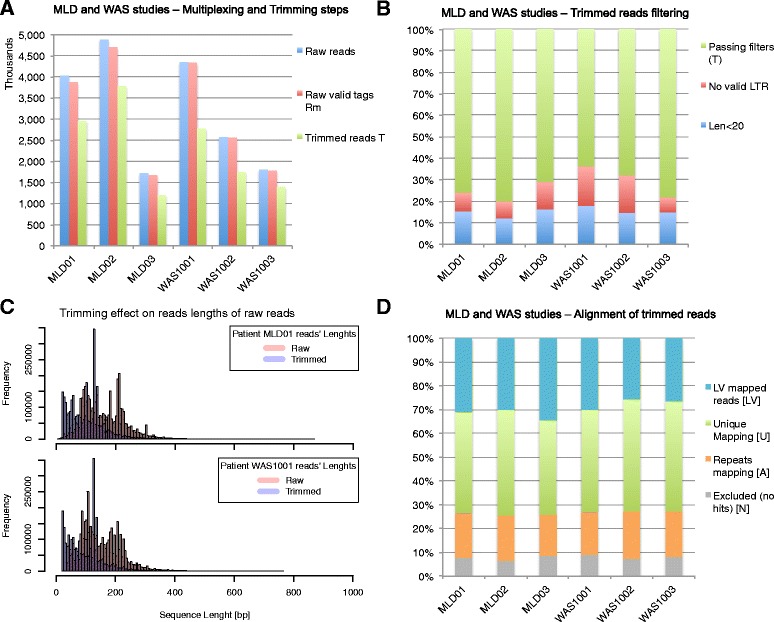
**Pipeline results for the MLD and WAS clinical trials. (A)** Histogram plot of sequence reads for each patient showing the decrease in absolute number of reads after the demultiplexing and trimming steps. **(B)** Bar plot of the relative percentage of raw reads passing the trimming step (in green) and filtered reads by length (blue bar) or invalid LTR reads (red bar) for each patient. **(C)** Effect on the frequency distribution of the length of raw reads after trimming for two datasets obtained from two GT patients (MLD01 and WAS1001). **(D)** Bar plot of the relative percentage of aligned reads for each patient: the input sequences are the trimmed reads, whereas the resulting subsets from the alignment step are LV, *U*, *A*, and *N*.

**Figure 6:**
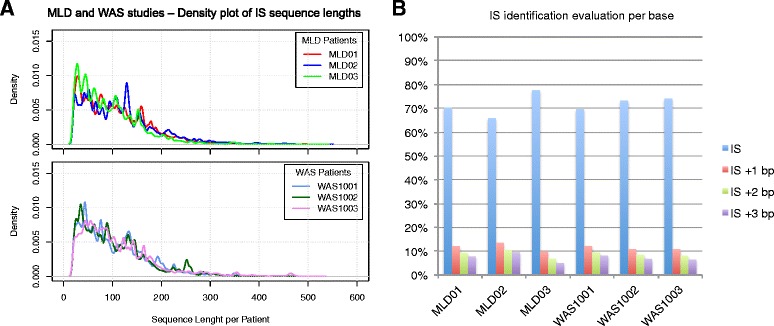
**IS reads and loci analysis. (A)** Distribution of trimmed IS reads for all patients in the MLD and WAS clinical trials. **(B)** Histogram plot of IS identification for each patient: values are in percentage and represent the amounts of covered bases within the 3 bp range for each IS.

The percentage of reads with correct barcodes ranged from 96.61% to 99.23% of the total (Figure 5A). On the other hand, about 30% of the sequencing reads was excluded after trimming (Figure 5B) due to the absence of a valid LTR (12% on average) or because they were too short to be mapped on the reference genome (15% on average). The decrease in number of reads associated to the first three filtering steps was comparable in all patients (Figure 5A, B). As shown in Figure 5C, the trimming step modifies the distribution of the length of sequencing reads by introducing a shift towards smaller sizes and a slight change in its profile. After the alignment to the reference genome (Figure 5D) about 40% of the initial reads was excluded from further analysis because: (1) lacking a valid match on the reference genome (6% on average); or (2) because these reads were vector-only sequences (21% on average); or (3) repetitive elements that could not be univocally mapped to the reference genome (13% on average). Reads left after quality-based filtering (the ***R*** dataset) were, on average, 90.87% of ***U*** reads, with a standard deviation of less than 1%. The sequence length distribution profile of the univocally mapped reads (the ***R*** dataset) was similar for all patients of both clinical studies (Figure 6A). Finally by applying the sliding window approach described above, we identified all IS reads that fall in the same 3 bp interval as belonging to the same integration event (the IS merging step), the number of such reads can be seen as a measure of the ‘signal power’ of the integration (Figure 6B). To evaluate the precision of this approach, we computed the percentage of IS positions (starting covered bases) hit by an IS read within each window: as shown in Figure 6B, over 60% of IS bases fall in the first position (blue bar), while for other bases the percentage decreases as the distance from the IS increases.

In summary, we found that four major steps account for the observed strong reduction in the amount of sequence reads: (1) LTR recognition; (2) short sequence reads; (3) vector-only sequences; (4) repetitive elements. To increase the yield of sequences with a recognizable LTR, further optimization of the parameters for its recognition could be adopted, carefully evaluating the possible impact on the alignment quality and errors. To significantly reduce the number of short length reads, optimization of the wet procedures could be required, such as developing other LAM-PCR protocols optimized for the amplification of long products as well as the use of sequencing technologies that allow the characterization of long fragments (that is, PacBio platform for single molecule real time sequencing). Similarly, to reduce vector-only sequences, further technological improvements should be adopted. In particular, the use of novel oligonucleotides for LAM-PCR amplification annealing in non-repeated portions of the HIV genome (thus avoiding the LTR) could drastically reduce or completely eliminate the presence of such non-informative contaminant.

The issue of the presence of repetitive sequences that cannot be mapped univocally to the reference genome is more complex to solve but it would lead to a major improvement of IS analysis procedures. Many of the analyses take into account the number of univocally mapped integration sites as surrogate markers of clonality, including the tracking of HSPC reconstitution and differentiation to estimate the polyclonal hematopoietic repertoire in terms of population diversity, as well as the number of active stem cells that reconstituted the hematopoietic system. Therefore the lack of precise information regarding the number of cell clones that harbor integrations in repetitive regions could have a detrimental impact on the overall picture of these analyses. To solve this issue, novel PCR techniques that increase the length of the sequence reads may be developed, thus increasing the mappability of PCR sequenced products [8].

## Conclusions

IS analysis is an essential step for assessing the safety and efficacy of molecular therapies that use genetically-modified hematopoietic stem cells via integrating viral vectors. Typically, for safety and efficacy studies, IS analyses should preferably be performed on different hematopoietic cell lineages that are purified and isolated from the bone marrow and/or peripheral blood of GT patients at different time points after transplantation. VISPA was extensively used to monitor lentiviral integrations in two clinical studies for the treatment of MLD and WAS, enabling to efficiently extract IS information from a large number of samples (N > 1,400) and sequencing reads (N = approximately 20 × 10e6 reads) and to verify the safety and efficacy of the treatments in various aspects [3,4]. For the first time, we were able to observe *in vivo* molecular patterns of human stem cell differentiation and proliferation, opening new perspectives to understand cell dynamics through population studies. Moreover, even if IS analysis has not been fully standardized yet, drug administration agencies require to perform IS analysis both in preclinical experimentations and GT clinical trials to identify potential insertional mutagenesis events. The emerging interest of the scientific community on these studies highlights the great potential of IS analysis and, consequently, of tools capable of efficiently and reliably perform the associated computational steps such as the one presented here. Hence, this further increases the importance of bioinformatics tools such as VISPA, designed not only for accuracy and efficiency, but also for usability by researchers without higher level of informatics expertise.

## Availability and requirements

- **Project name**: VISPA
- **Project home page**: https://github.com/crs4/vispa
- **Operating system(s)**: Unix
- **Programming language**: Python
- **Other requirements**: Galaxy, Hadoop
- **License**: GNU GPLv3
- **Any restrictions to use by non-academics**: commercial use is permitted, see the GPLv3 for requirements

## Additional files

Additional file 1:
**Generation of the test set and accuracy evaluation setup.** This document provides in-depth details on the generation of the *in silico* dataset, and describes the experimental setup used to assess the accuracy of VISPA and other three IS analysis tools (MAVRIC, SeqMap and QuickMap). See http://genomemedicine.com/content/supplementary/s13073-014-0067-5-s1.pdf. Additional file 2:
***In silico*** **dataset sequences and accuracy assessment results.** This is a Microsoft Excel file with two tabs, ‘FASTA formatted sequences’ and ‘comparison’: the former contains the 455 sequences that make up the synthetic dataset in FASTA format, while the latter provides detailed information and experimental results for each sequence. Specifically, the ‘INPUT’ column group contains sequence annotation; the ‘BWA’ and ‘GEM’ column groups list results for each aligner; in the remaining columns, results are provided for VISPA and the other three tested tools. See http://genomemedicine.com/content/supplementary/s13073-014-0067-5-s2.xlsx. Additional file 3:
**Analysis of mismatched IS.** **(A)** Box plot of the distances, in terms of genomic position (bp), between each mismatched IS and the reference IS, for MAVRIC, SeqMap, and QuickMap. **(B)** Total number of mismatched ISs for different bp intervals. See http://genomemedicine.com/content/supplementary/s13073-014-0067-5-s3.pdf.

## Abbreviations

API: Application programming interface
FDR: False discovery rate
GT: Gene therapy
GUI: Graphical user interface
HSPC: Hematopoietic stem/progenitor cell
IS: Integration site
LAM-PCR: Linear amplification mediated polymerase chain reaction
LV: Lentiviral vector
MLD: Metachromatic leukodystrophy
NGS: Next-generation sequencing
WAS: Wiskott-Aldrich syndrome
γ-RV: γ-retroviral vector
