# Supplementary material for: VISPA: a computational pipeline for the identification and analysis of genomic vector integration sites
Source: Genome Med. 2014 Sep 3;6(9):67. doi: 10.1186/s13073-014-0067-5 (PMC4169225; doi:10.1186/s13073-014-0067-5)
Supplement: Additional file 1: — Generation of the test set and accuracy evaluation setup. This document provides in-depth details on the generation of the in silico dataset, and describes the experimental setup used to assess the accuracy of VISPA and other three IS analysis tools (MAVRIC, SeqMap and QuickMap). See http://genomemedicine.com/content/supplementary/s13073-014-0067-5-s1.pdf. [file 13073_2014_67_MOESM1_ESM.pdf]

VISPA: a Computational pipeline for the identification and analysis of genomic vector integration sites

Andrea Calabria[1,*], Simone Leo[2,3,*], Fabrizio Benedicenti[1], Daniela Cesana[1], Giulio Spinozzi[1,4], Massimilano Orsini[2], Stefania Merella[5], Elia Stupka[5], Gianluigi Zanetti[2¥], Eugenio Montini[1¥§]

[1]San Raffaele Telethon Institute for Gene Therapy (TIGET), San Raffaele Scientific Institute, 20132 Milano, Italy.

[2]Center for Advanced Studies, Research and Development in Sardinia (CRS4), 09010 Pula (CA), Italy.

[3]Università degli Studi di Cagliari, 09124 Cagliari, Italy.

[4]Department of Informatics, Systems and Communication (DISCo) - University of Milano-Bicocca. Milano, Italy

[5]Center for Translational Genomics and Bioinformatics, San Raffaele Scientific Institute, Via Olgettina 58, 20132, Milano, Italy

** These authors equally contributed to this work*

[¥]*Co-senior Authors*

[§]*Corresponding Author*

Additional file 1

Test set generation

To create a simulated dataset of integration sites (IS) we selected 283 genomic locations (UCSC human reference genome hg19 GRCh37, source UCSC http://hgdownload.soe.ucsc.edu/goldenPath/hg19/bigZips/) representing three categories of IS:

1. 120 randomly selected genomic locations with a comparable number of loci for each chromosome, obtained by exploiting a random number generator provided by MS Excel in the range of each chromosome length.
2. 132 genomic locations characterized by sequences with low complexity or containing repetitive elements with potentially multiple matches in the genome. These sequences were semi-randomly selected by querying the repeat masker annotation dataset available at UCSC so that each sequence annotated as "repeat" is represented around three times.
3. 31 IS randomly selected from a patient of the MLD clinical study (Biffi, Montini et al., 2013 – Science).

The genomic distribution of the loci along the chromosomes is shown below.

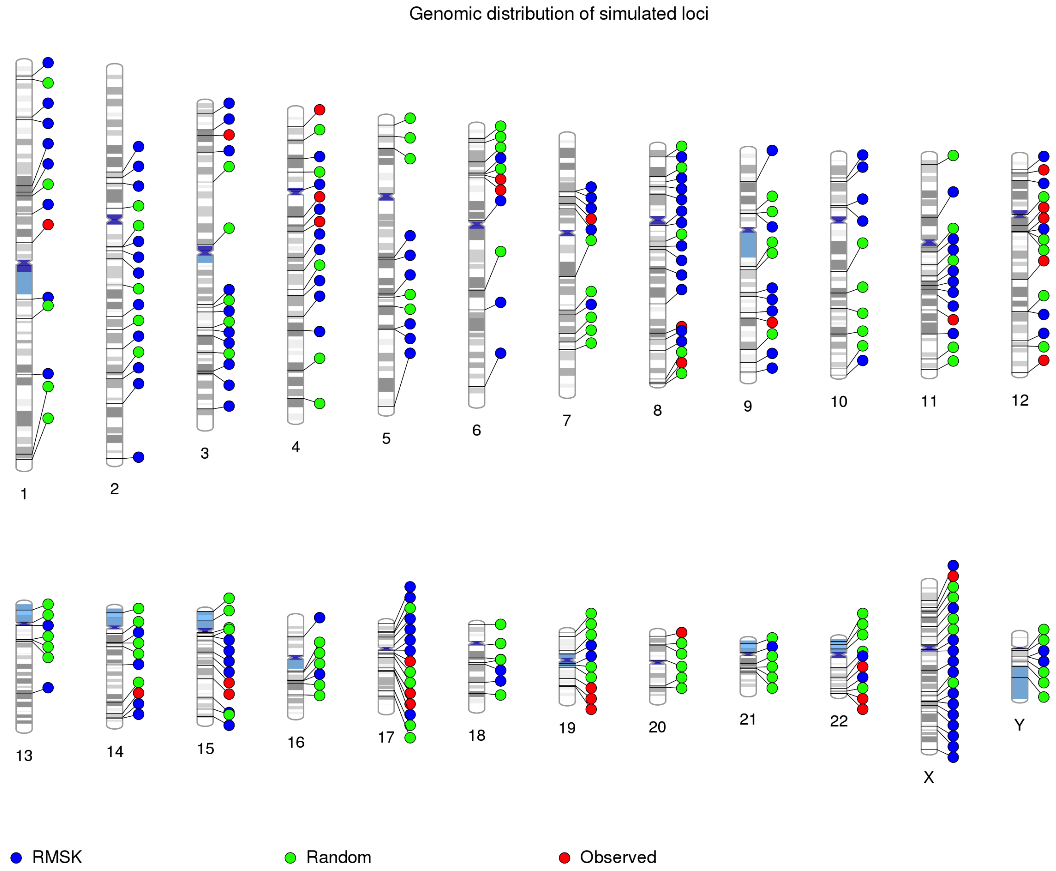

Genomic distribution of the 283 randomly selected loci.

Chromosomal distribution of input IS where each point represents a locus in the genome, with different colors used for each category: "RMSK" (from the repeat masker DB), "Random" (randomly selected from the genome) and "Observed" (from the MLD study).

Given that real IS datasets are constituted by DNA fragments containing the vector LTR flanked by genomic sequences digested with a restriction enzyme to allow the ligation of a linker cassette (LC), we extracted the nucleotide sequence flanking each genomic location (up to 3kb) in both directions and kept only the sequences between the randomly generated genomic position and the first Tsp509I restriction site (AATT). Only sequences with ≥20bp were kept for further analysis, leading to a total of 455 genomic intervals. The sequence length distribution of the remaining 455 genomic intervals is depicted below.

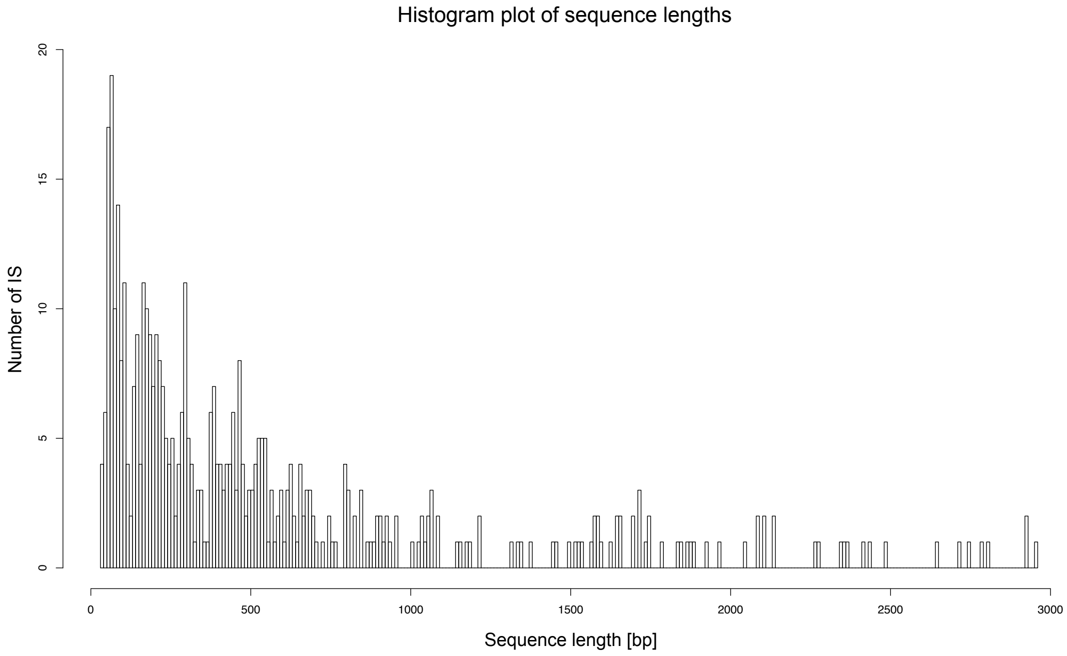

Sequence length distribution of 455 selected genomic intervals.

The following sequence logo plot shows that the information content of the generated dataset does not present any significant biases (no conserved k-mers, no motifs, etc.).

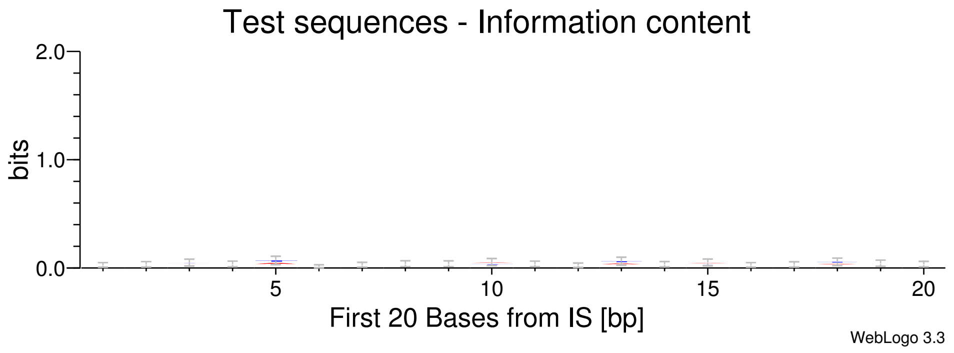

Logo-plot representation of the information content for the input dataset.

For each position, the plot shows a stack of five symbols (A, C, G, T and N), where the overall height represents the sequence conservation at that position, while the height of each symbol of the stack indicates its relative frequency at that position. The plot was generated with WebLogo (http://weblogo.berkeley.edu/).

Finally, to the selected sequences we added the LTR sequence in correspondence of the randomly selected the genomic location and the LC sequence in correspondence of the first Tsp509I restriction site (see Additional File 2 for the full list of *in silico* generated IS).

Since all tested tools except VISPA are accessed via a web service with limited computational power, we used a relatively small dataset in order to perform the evaluation within a reasonable time.

Evaluation of VISPA and other IS analysis tools

VISPA was tested on an *in silico* dataset of genomic integration sites (described in Additional files 1 and 2). Results were compared to those obtained with other three available tools for integration site analysis tools: Mavric [32], SeqMap [33] and QuickMap [34]. Two well established sequence aligners were used to perform the tests: BWA [35] and GEM [36].

Each tool was run using the same input test dataset and reference genome with the following exceptions: since SeqMap does not contain the reference genome HG19, we exploited Batch Coordinate Conversion (liftover) available at UCSC to convert the genomic coordinates from HG18 to HG19 (Human Genome, version 2009); for QuickMap, BWA MEM and GEM the input we used was the FASTA file of genomic sequences without LTR and LC.

Results of the four IS tools are summarized in Additional file 2.
