# Supplementary figures and images for: VISPA: a computational pipeline for the identification and analysis of genomic vector integration sites
Source: Genome Med. 2014 Sep 3;6(9):67. doi: 10.1186/s13073-014-0067-5 (PMC4169225; doi:10.1186/s13073-014-0067-5)

**A**

### Mismatch comparison among IS tools

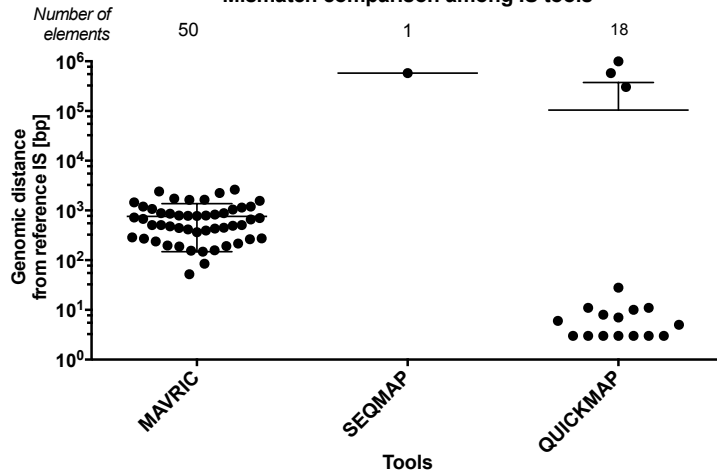**B**

### Mismatches Analysis

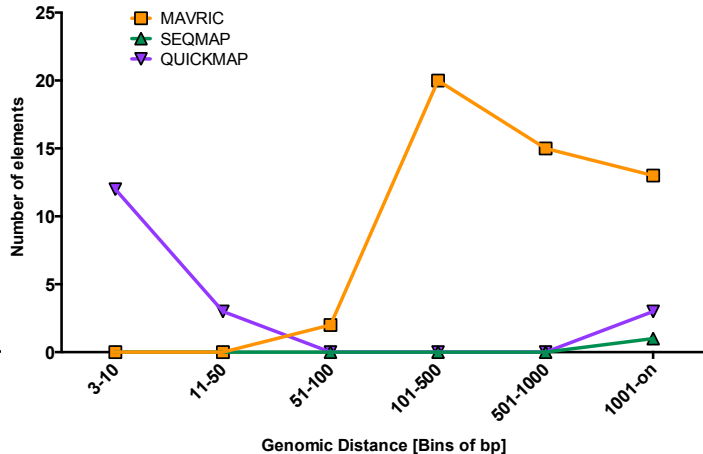

Supplement: Additional file 3: — Analysis of mismatched IS. (A) Box plot of the distances, in terms of genomic position (bp), between each mismatched IS and the reference IS, for MAVRIC, SeqMap, and QuickMap. (B) Total number of mismatched ISs for different bp intervals. See http://genomemedicine.com/content/supplementary/s13073-014-0067-5-s3.pdf. [file 13073_2014_67_MOESM3_ESM.pdf]
